
JOURNAL INFORMATION
==============================
NLM Title Abbreviation: ScientificWorldJournal
Journal ID: ScientificWorldJournal
NLM Journal ID: 101131163
Journal ID: TSWJ

The Scientific World Journal

ISSN: 2356-6140
EISSN: 1537-744X
Publisher: Wiley

ARTICLE INFORMATION
==============================
PMCID: PMC5956511
PMID: 15349530
DOI: 10.1100/tsw.2004.49
Article ID: 256830
Article version: 1
Subjects: Research Article

Endourologic Management of Upper Tract Transitional Cell Carcinoma

Clark Peter E.
Streem Stevan B.
Cleveland Clinic Foundation, Department of Urology, Cleveland, Ohio, USA
Academic Editor: Anthony Atala

Publication date: 2004
Electronic publication date: 2004 Jun 7
Volume: 4
First page: 62
Last page: 75
Copyright: Copyright © 2004 Peter E. Clark and Stevan B. Streem.
Copyright year: 2004
License: This is an open access article distributed under the Creative Commons Attribution License, which permits unrestricted use, distribution, and reproduction in any medium, provided the original work is properly cited.
License URL: https://creativecommons.org/licenses/by/3.0/

==============================
Article
*TheScientificWorldJOURNAL* (2004) 4 (S1), 62–75
ISSN 1537-744X; DOI 10.1100/tsw.2004.49

### Endourologic Management of Upper Tract Transitional Cell Carcinoma

Peter E. Clark, M.D. and Stevan B. Streem, M.D.
*Cleveland Clinic Foundation, Department of Urology, Cleveland, OH*

**E-mails**: peclark@wfubmc.edu

*Previously published in the Digital Urology Journal*

**DOMAIN**: urology

### INTRODUCTION

Transitional cell carcinoma (TCC) occurs only rarely in the upper urinary tract and accounts for less than 5% of all cases of urothelial neoplasia.[1] Like TCC of the bladder, however, it often represents a field change disease characterized by multiple recurrences in both time and space, though this tendency towards polychronotopism is generally confined to the ipsilateral renal unit or to the bladder. As a result, the incidence of TCC in the contralateral renal unit is only 3%.[2,3] This natural history supports the use of nephroureterectomy with resection of a cuff of bladder as the gold standard for the management of upper urinary tract TCC. It in fact attempts to perform more conservative, renal sparing open operations for upper tract TCC that have universally met with high rates of local or ipsilateral recurrence. [4-6]

With the advent of sophisticated techniques for the endourologic management of many benign urologic diseases of the upper tracts, there has been growing enthusiasm for the application of these same techniques to the management of upper tract TCC. Recent advances in the fields of retrograde ureteroscopy and percutaneous pyeloscopy include the development of small caliber, actively deflecting fiberoptic telescopes, improved optics, and the development of small caliber adjunctive instruments and laser fibers.[7-9] These advances have opened the field of upper tract TCC to a variety of innovative approaches, particularly for those patients in whom standard nephroureterectomy may present a prohibitive operative risk or leave the patient functionally anephric. These include patients with upper tract TCC in an anatomically or functionally solitary kidney, those with bilateral disease, or patients with significant chronic renal insufficiency. Endourologic alternatives to open surgery have also been advocated for those patients with significant comorbid disease in whom an open operation would be poorly tolerated.

This article will review the endourologic management of upper tract TCC to further help define its role in current urologic practice.

### DIAGNOSIS AND STAGING

Transitional cell carcinoma of the upper urinary tract will often manifest itself as a filling defect on excretory urography, though a number of benign lesions can present with this same finding. These include radiolucent stones, blood clots, sloughed renal papillae, extrinsic vascular compression, pyelitis, ureteritis cystica, tuberculosis, and endometriosis.[10] As the management of each of these disease processes clearly differs from that of upper tract TCC, it is important to obtain an accurate diagnosis before a treatment regimen can be developed and initiated (Figure 1).

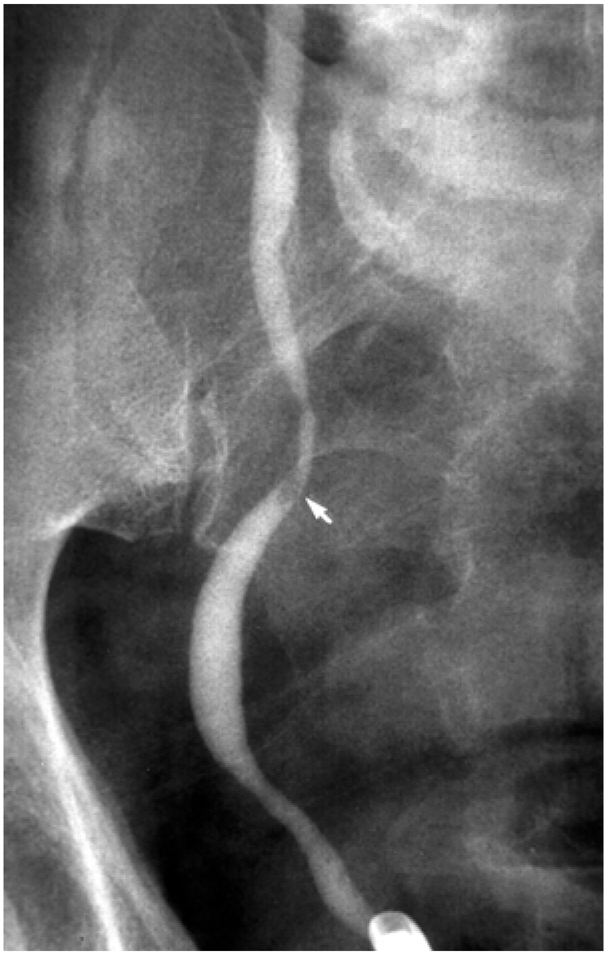

**FIGURE 1A.** Retrograde studies reveal a small filling defect in the distal right ureter (arrow). This patient has nephrotic syndrome and requires "nephron sparing" management.

The standard evaluation for a filling defect seen on excretory urography includes the use of voided urine cytology, cystoscopy with retrograde pyelography, and selective upper tract urine cytology. Retrograde pyelography mainly serves to confirm the presence of a filling defect. Cytology can be very useful in cases of high grade TCC or carcinoma in situ (CIS). For CIS of the upper tract, selective cytology has been reported to have an accuracy rate as high as 80%.[11] For low grade lesions (grade 1), however, voided urine cytology can have a false negative rate as high as 96%, and even selective cytology in the setting of well differentiated tumors has an accuracy rate of only 10 to 40%.[11-14] Retrograde brush biopsy has also been used but this again can have a false negative rate of 16%.[15,16] Other imaging modalities such as computerized tomography (CT) or ultrasound (US) are useful in distinguishing calculus disease, but otherwise have limited utility in diagnosis.

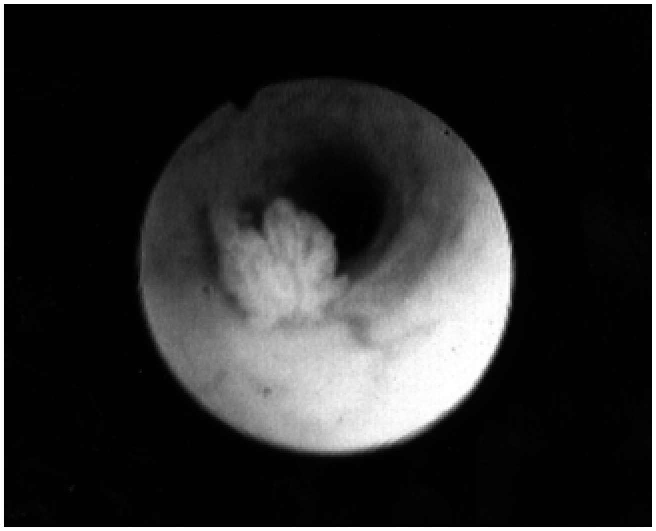

**FIGURE 1B.** Ureteroscopic view of low grade, low stage polypoid TCC. Definitive management was accomplished with ureteroscopic biopsy and fulguration.

The addition of ureteropyeloscopy to the evaluation for an upper urinary tract filling defect can greatly enhance the urologistsí diagnostic accuracy and overcome many of the shortcomings of the standard diagnostic regimen and several studies have now demonstrated its utility. In a study by Streem et. al., patients with upper tract filling defects underwent a standard diagnostic evaluation including excretory urography, cystoscopy, retrograde pyelography, voided and selective urinary cytology, and, in some cases, flouroscopically guided brush biopsy.[17] CT scan or ultrasonography was used for selected cases. This standard regimen yielded the correct diagnosis in 58% of cases. In contrast, the addition of rigid ureteropyeloscopy to these same patients increased the diagnostic accuracy to 83%. In that study, diagnostic failure was usually secondary to inaccessibility of the lesion. With the advent of smaller and flexible instrumentation, the accuracy of diagnosis has improved even further, such that a diagnostic accuracy of 90 to 100% can now be achieved in experienced hands.[18,19] As a result, ureteropyeloscopy has clearly become a valuable standard addition to the urologistsí armamentarium.

A further advantage of ureteropyeloscopy is that it offers the opportunity to biopsy any lesions encountered during the procedure thus allowing for pathologic confirmation of the disease process. For TCC, it also allows for the determination of tumor grade and, in some cases, stage. A recent series of 51 patients from Thomas Jefferson University undergoing ureteroscopic biopsy followed by open nephroureterectomy found 90% agreement between grade on ureteroscopic biopsy and grade on the final pathologic specimen for low or moderately differentiated tumors, and 92% agreement for poorly differentiated tumors.[20]

In addition to grade, determination of stage is important in determining the feasibility of renal sparing surgery. Several reports have shown the limitations of CT scans in differentiating invasive versus noninvasive disease.[21-23] A study by McCoy et. al. determined the sensitivity and specificity of CT scan in determining parenchymal invasion as 75% and 43% respectively.[23] The sensitivity and specificity for fat invasion was 67% and 44% respectively. Badalament et. al. demonstrated that with definite evidence of tumor extension beyond the wall of the ureter or renal pelvis, CT scan was accurate in 100% of cases.[21] However, in the absence of frank extension beyond the wall of the ureter or renal pelvis, CT scan failed to identify invasive disease in 59% of their patients. It has been suggested that ureteropyeloscopic biopsy is also unreliable in determining stage.[7, 24] This is secondary to the thin wall of the ureter and pelvis and the need to avoid perforation and loss of integrity of the urinary tract in the setting of TCC. Nevertheless, several studies have suggested a good correlation between the grade of TCC and the stage of the tumor.[13,20,25,26] Murphy et. al. found that 47 of 49 (96%) patients with grade one TCC also had Stage 1 disease.[13] Heney et. al. reviewed 60 patients who underwent surgery for upper tract TCC and found 9 with grade 1 tumors, all of whom had superficial disease.[25] Similarly Chesko et. al. reviewed 29 nephroureterectomy cases and found 7 patients who had grade 1 disease, all of whom had superficial non-invasive tumors.[26] As such, the combination of low grade on biopsy and absence of frank extension outside of the urinary tract by CT scan clearly suggests the disease is likely to be superficial.[27,28]

Even with modern advances in instrumentation, situations may occasionally arise in which retrograde ureteropyeloscopy is not possible. This occurs not infrequently in patients who have had a prior urinary diversion in whom locating and negotiating the uretero-intestinal anastomosis in a retrograde fashion may be impossible. In this circumstance, if a standard diagnostic evaluation of an upper tract filling defect remains indeterminate, then consideration should be given to antegrade pyeloscopy or ureteroscopy via a percutaneous approach as discussed later in this paper.[29]

### DEFINITIVE TREATMENT WITH URETEROSCOPY

Initial results with the ureteroscopic management of upper tract TCC have been cautiously encouraging. Gerber and Lyon reviewed the literature in 1993 and found that in 51 patients managed ureteroscopically, 7 developed a local recurrence (13.7%) at a follow-up of 2 to 67 months.[28] Such results have now been further supported by more recent reports with longer follow-up. Martinez-Pineiro et. al. recently reported on 28 patients treated for upper tract TCC with ureteroscopy alone.[30] The overall follow-up in their entire group was 2 to 119 (mean 30.6) months during which there were 8 recurrences (28.5%). The overall cause specific mortality in their group of patients treated endourologically was 13.6%. Elliot et. al. reported on their long term follow-up on 44 patients treated endourologically for upper tract TCC including 37 patients treated with ureteroscopy and 7 patients treated percutaneously.[31] They reported a 38.6% local recurrence rate with a mean follow-up of 5 years (range 3 months to 11 years). Only 6 patients (14%) ultimately required nephroureterectomy and only one recurrent tumor progressed in stage during that time, while none had progressed in grade. Of note, their series had one patient with a recurrence after 64 months of follow-up, which emphasizes the need for long-term surveillance in this patient population. A more recent review found a similar recurrence rate among 14 studies. The overall recurrence rates among these studies was 33% for 61 renal pelvic tumors and 31.2% for 144 ureteral tumors managed endourologically.[27] Recurrent bladder tumors occurred in 43.2%, again emphasizing the need for continued cystoscopic surveillance in these patients.

Although initial experience with the endourologic management of upper tract urothelial tumors made use of electrocautery and fulguration of tumors, more recent work has emphasized the potential utility of lasers in the treatment of upper tract TCC. Schneller and Hofstetter reported 3 recurrences among 16 patients treated using the neodymium: yttrium-aluminum-garnet (Nd:YAG) laser with a mean follow-up of 13.8 months.[32] Schilling et. al. reported on one recurrence among 13 treated tumors using the Nd:YAG laser after a mean follow-up of 23 months.[33] Carson had no recurrences in 8 patients followed for 6 to 40 months.[34] Complete mucosal healing has been demonstrated after Nd:YAG laser fulguration after three weeks.[33]

The Nd:YAG laser is used predominantly for deep tissue ablation and has a depth of penetration of 5 to 6 mm. The laser fiber functions best when not in contact with the tumor to avoid charring of the tip. Care must be taken, as studies by Smith et. al. have shown, that use of the Nd:YAG laser at 35 watts for 2 seconds caused transmural necrosis of the ureter in dogs.[35] Most authors, therefore, recommend use of the Nd:YAG laser at 20 watts for one second.[27, 36] Treated tumor must continually be removed during the procedure to allow for exposure and treatment of deeper layers.

The Holmium: yttrium-aluminum-garnet (Ho:YAG) laser has also recently been advocated for use in the urinary tract.[37, 38] It has a tissue penetration depth of only 0.5 mm, such that the visualized effect during endoscopy represents the maximal depth of tissue penetration. The advantage of the Ho:YAG laser lies in its ability to provide superficial ablation in the thin walled ureter as well as the ability to greatly aid in coagulation and homeostasis. It has also been advocated for use in ablating and opening the lumen of an occluding ureteral neoplasm.[27] The Ho:YAG laser should be used with the tip of the laser fiber in contact or very close to the tissue to be ablated. A recent report by Keeley et. al. reported good results using a combination of Nd:YAG laser to ìdebulkî the majority of the tumor, followed by the Ho:YAG laser for more superficial ablation and coagulation in 26 of 49 kidneys.[39] Of 41 renal units treated only ureteroscopically, there were 8 recurrences (19.5%) after a mean follow-up of 35.1 months. Not unexpectedly, grade 2 or higher disease, size greater than 1.5 cm., and multifocal disease all were correlated with higher recurrence rates. Interestingly, 6/8 recurrences (75%) were missed radiographically and picked up only on routine ureteroscopic surveillance. This suggests a role for regular ureteroscopic surveillance as part of the follow-up for select patients with upper tract TCC treated with nephron sparing techniques.[27, 39]

The use of argon photoirradiation for the treatment of ureteral TCC has also been reported.[40] Johnson et. al. reported on three patients with low-grade ureteral TCC treated with argon photoirradiation with no recurrences at 5, 7, and 22 months. Further experience with this techniques is required before it is used routinely.

### COMPLICATIONS OF URETEROSCOPY

Potential complications of ureteroscopic diagnosis and treatment of upper tract urothelial tumors include perforation of the ureter and stricture formation, and in this setting the rate of perforation of the ureter/renal pelvis has been reported to be 0% to 10.2%.[30,31,39] In all cases, this was successfully managed conservatively with either a ureteral stent or nephrostomy tube and no adverse sequelae have been reported.

The reported stricture rate in large series varies between 4.9 to 13.6%.[30,31,39] These strictures were treated by either prolonged ureteral stent placement, or endoscopic management of the stricture with incision or balloon dilation, and short-term stent placement.[30,31] It has been suggested that the use of lasers will decrease the stricture formation rate. For example, Schmeller and Hofstetter had only one stricture among 8 patients treated with Nd:YAG laser alone versus three among 8 patients treated using a combination of Nd:YAG laser and electroresection.[32] Similarly Keeley et. al. found no strictures among 22 patients who underwent treatment with the Ho:YAG laser.[39] This has been attributed to the minimal tissue penetration of these lasers.[27] However, in contrast to these studies, Martinez-Pineiro et. al. found no appreciable difference in their patients treated by laser versus electroresection and concluded that lasers offered no significant benefit.[30]

One of the theoretical objections to endoscopic management of upper tract malignancy is the potential for endoscopy to cause local seeding of tumor and/or subsequent extraluminal local recurrence or dissemination. These concerns were highlighted by Tomera et. al. in a study in which intraoperative pyeloscopy was performed during open exploration of the kidney for indeterminate renal pelvic filling defects.[41] The authors noted local recurrence in the renal fossa in 2 of 18 patients. These occurred at 6 and 36 months respectively and were associated with what had initially been grade 1 or grade 2, noninvasive lesions. Those authors had raised concern that the pyeloscopy had caused extraluminal seeding of the tumor. There is also at least one case report of tumor in the lymphatic and vascular structures of the submucosa in a nephroureterectomy specimen removed immediately after ureteropyeloscopy.[42] The authors proposed that this represented seeding from the ureteroscopic procedure, which had been performed using a pneumatic cuff to pressurize saline irrigation to 200 mm Hg.

In contrast to these studies, McCarron et. al. performed 33 open pyeloscopies without any instances of extraluminal recurrence.[43] Similarly, Kulp et. al. performed immediate nephroureterectomy after ureteropyeloscopy in 13 patients and found no evidence, on pathologic sectioning, of extraluminal tumor implantation or seeding.[44] In addition, in cases of perforation during endoscopic management of upper tract TCC, no adverse sequelae have been noted.[30,31,39] It is generally felt that the risk of extraluminal seeding of TCC from ureteroscopy is, at most, very small.

Andrews and Segura reported on an unusual complication of endoscopic management. They discussed a case of intrarenal pelvic explosion during endoscopic management of upper tract TCC.[45] This can be avoided by minimizing the amount of continuous coagulation, and subsequent intraluminal hydrogen formation, during the procedure.

### PERCUTANEOUS MANAGEMENT

Although ureteroscopic management of upper tract TCC has the theoretic advantage over percutaneous management in that the integrity of the urothelium is maintained, the percutaneous approach offers several practical advantages over ureteroscopy. These include superior visibility and access to renal pelvic tumors in most cases, availability of larger instrumentation, and access to virtually any renal unit irrespective of prior operative procedures. Thus, patients with ureteroscopically difficult to access infundibulocalyceal tumors, or large renal pelvic tumors (greater than 1.5 cm.), or those patients in whom prior surgery such as an a urinary diversion makes retrograde ureteroscopic access difficult, are generally best treated using the percutaneous approach when nephron sparing management is warranted (Figure 2).

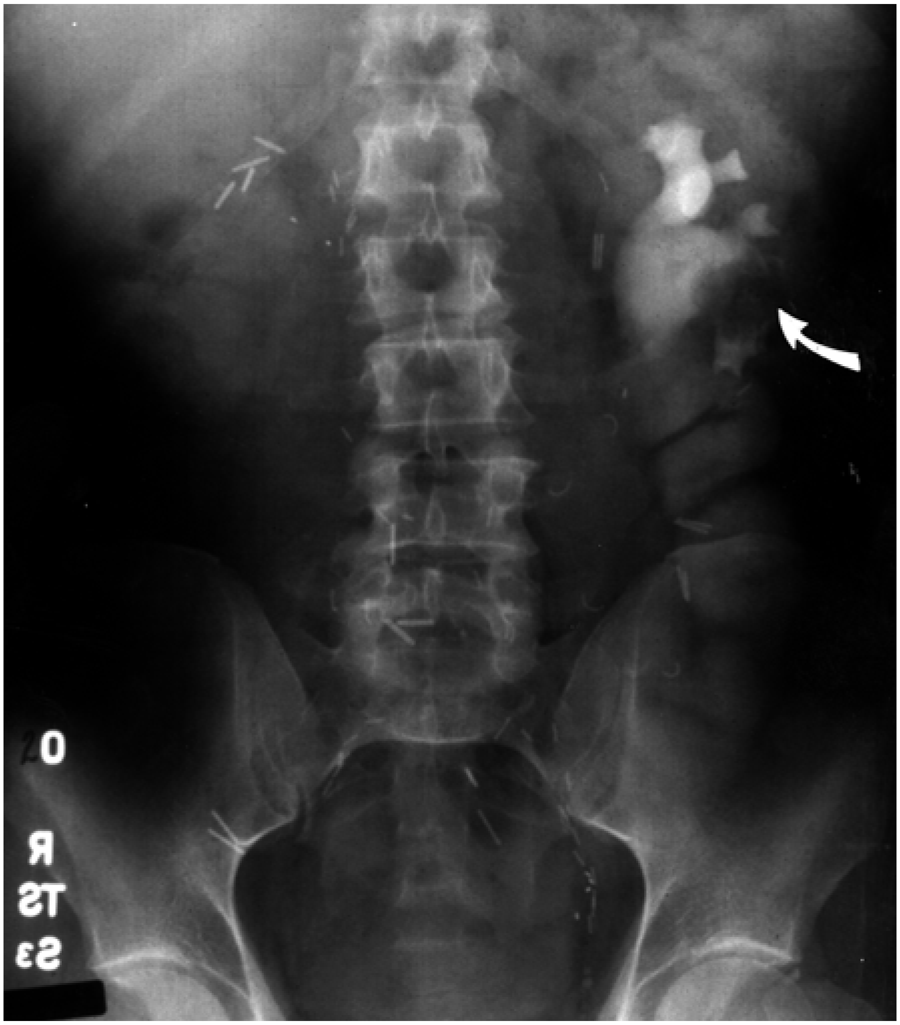

**FIGURE 2A.** Intravenous urogram in this patient who had previously undergone a right nephroureterectomy and left ureteral resection with ileal interposition suggests a 3 cm filling defect (arrow) in the left lower infundibulocalyx.

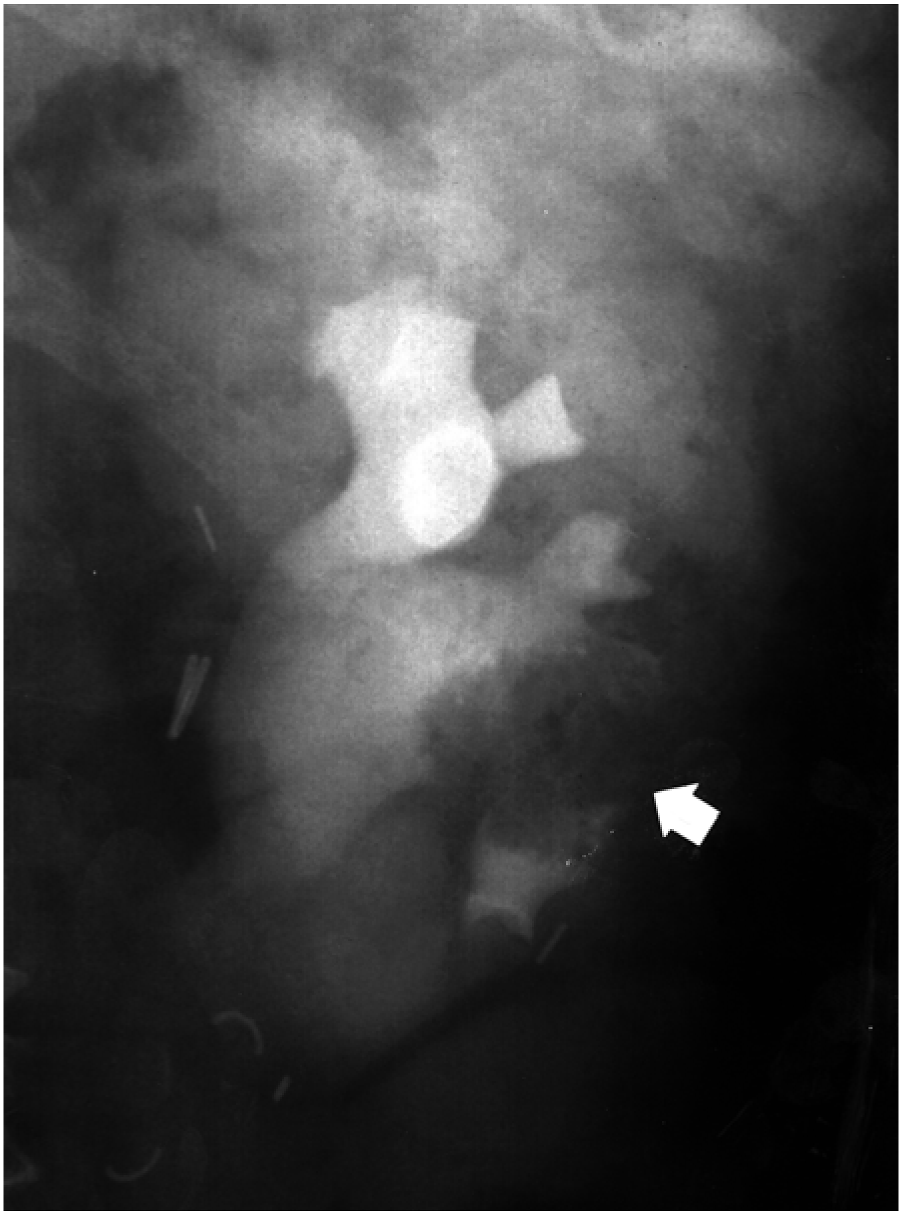

**FIGURE 2B.** Tomographic view confirms a 3 cm. irregular filling defect (arrow) consistent with recurrent TCC.

The initial experience with percutaneous management of upper tract TCC was summarized by Gerber and Lyon who reviewed an early experience from six different institutions. [28, 46-51] The recurrence rate among these reports ranged from 0% to 45% at a follow-up of 4 to 45 months. Overall, there were 6 recurrences in 23 renal units for an overall recurrence rate of 26.1%. At least one of the studies found higher grade, multifocal disease and size over 2 cm. to be predictive of higher local recurrence rates.[48] Long-term follow-up and greater experience at many of these institutions has confirmed the safety and long-term efficacy of percutaneous management of upper tract TCC in carefully selected patients.[31,52-55] Vasavada et. al. updated an initial experience with 6 patients treated percutaneously, including the adjuvant use of topical bacillus calmette-guerin (BCG), for upper tract TCC at the Cleveland Clinic and found no local recurrences with a mean follow up of 22 months.[52] However, two patients in their series with a prior history of invasive TCC elsewhere in the urinary tract developed distant metastases. Jarrett et. al.

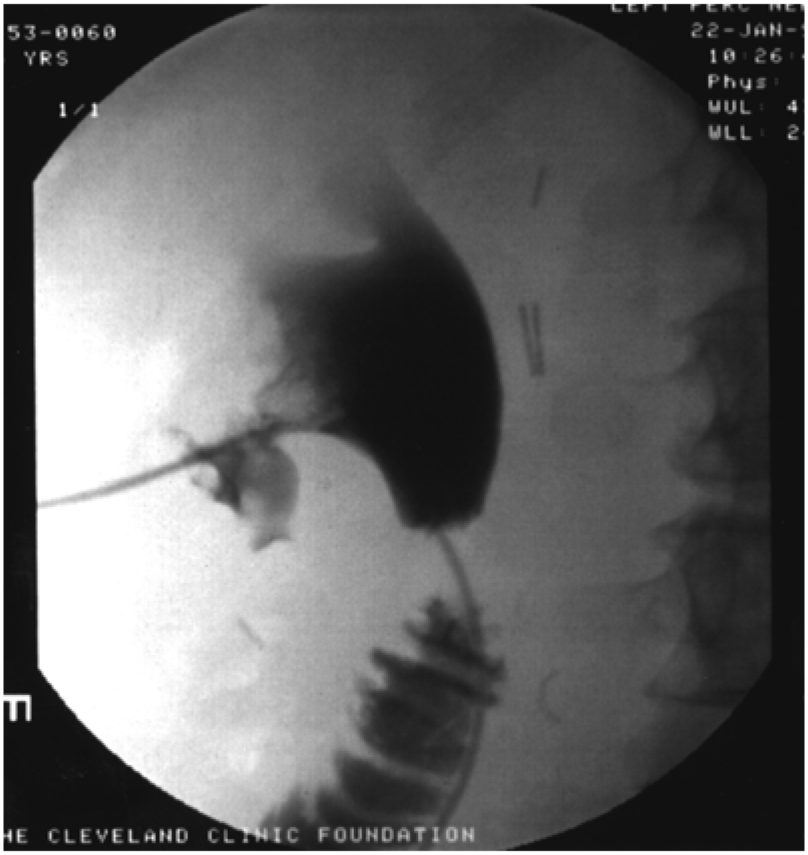

**FIGURE 2C.** Fluoroscopic view of percutaneous access in preparation for percutaneous electroresection of the tumor.

reviewed their experience with 36 renal units with an average follow up of 55.4 (range 9 to 111) months.[53] Their recurrence rates stratified by grade were 18% for grade 1/3, 33% for grade 2/3 and 50% for grade 3/3. All 4 cancer deaths in that series were in patients with grade 3 disease. Patel et. al. updated their experience with 26 patients undergoing percutaneous management.[54] They found local recurrence in 6/26 (23%) and bladder recurrence in 42%. As with ureteroscopic management, these bladder recurrences again highlight the need for ongoing cystoscopic surveillance in these patients. The calculated three year local recurrence-free survival rate was 86%, and the three year cause specific survival was 91%. Other centers have also had good results with local recurrence rates of 0% to 11.1% at a mean follow-up of 24 to 30.6 months.[30,56]

As with ureteroscopy, the Nd:YAG laser in percutaneous management of upper tract TCC is being used with increasing frequency, and a number of centers have now used this modality with a success rate at least comparable to electroresection.[28,31,53,56] In contrast, other investigators have had less enthusiasm for laser resection, and found higher recurrence rates when lasers were used.[30] The ultimate role for lasers in the endourologic management of upper tract TCC still remains to be determined.

Nakada and Clayman reported another adjunctive modality for the percutaneous management of upper tract urothelial tumors using either the rollerball electrode or the VaporTrode to percutaneously vaporize the tumor. [57] The initial safety and efficacy of this technique was demonstrated, but long term follow-up will be necessary to assess its true utility in this setting.

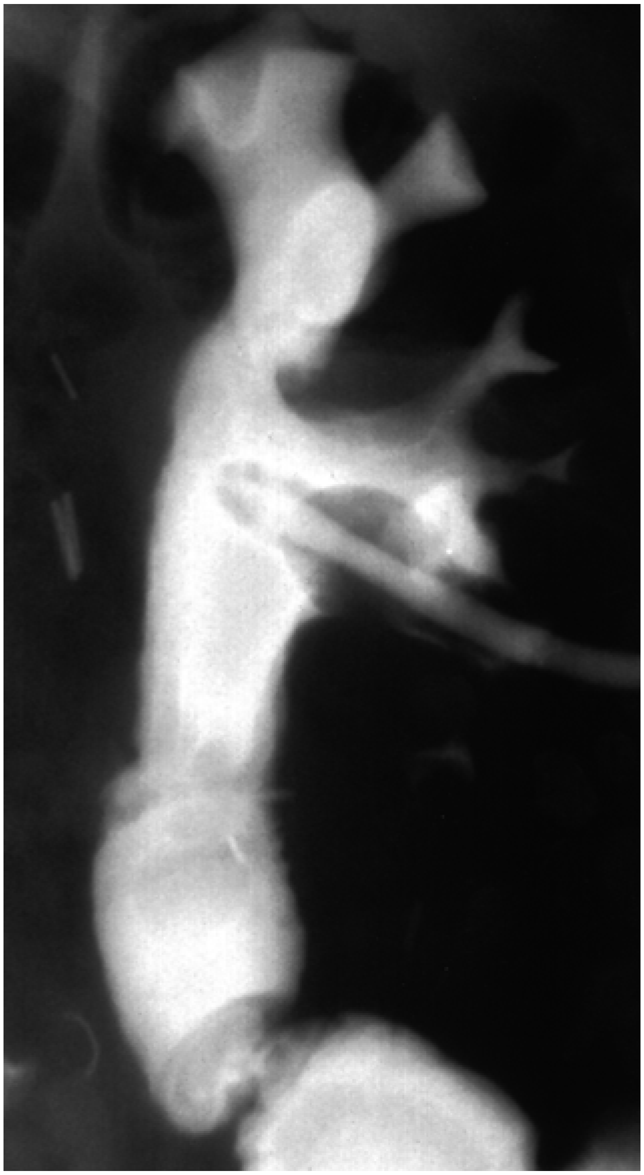

**FIGURE 2D.** Nephrostogram 48 hours after percutaneous resection reveals no residual tumor. There was no obstruction or extravasation, and a six weekly course of percutaneous BCG was begun 2 weeks later.

### COMPLICATIONS OF PERCUTANEOUS MANAGEMENT

The major theoretical concern with percutaneous management of upper tract TCC is the violation of the urothelial lumen with the hypothetical risk of extraluminal seeding, particularly along the nephrostomy tube tract. There has been at least one reported case of seeding of a nephrostomy tube tract by high-grade TCC.[58] It should be noted, however, that this case involved a diagnostic endoscopy in which the tumor was not resected at the time of the procedure and no adjuvant therapy was given. In all the reported series in which the primary tumor was resected at the same time the nephrostomy tract was established there have been no reported cases of nephrostomy tube tract seeding.[28,30,31,46-57] Several steps can be taken to lessen the likelihood of access tract seeding. These include single stage percutaneous access, tract dilation and tumor resection, and maintaining a low intrarenal pelvic pressure both through the use of a 30-F working sheath and maintaining the irrigation solution less than 40 cm above the level of the patient.[59]

Some European investigators also routinely use irradiation of the access tract either with an iridium wire or a commercial high dose rate radiation delivery system.[49,54] It has been suggested that sterile water should be utilized as the irrigant in these procedures for its cytolytic effect.[36] However, others argue that the risk of possible dilutional hyponatremia secondary to absorption is too high, and as such recommend saline during nephroscopy and glycine during electroresection.[59]

Other complications of percutaneous management are analogous to those encountered in the percutaneous management of renal calculi. These include the potential for hemorrhage, infection, hydrothorax, colonic perforation, and extravasation. Among 73 reported cases of percutaneous management of upper tract TCC, there were a total of 4 cases of hydrothorax secondary to attempted access via an upper pole infundibulum.[30,31,53,54] One case of colonic perforation was reported which was successfully managed with a parenteral diet and indwelling ureteral stent.[30] A total of four cases of extravasation were reported among these series with two cases of water absorption and dilutional hyponatremia treated successfully by furosemide and saline infusion. In one case with significant extravasation, a persistent urinary cutaneous fistula developed after adjuvant access tract irradiation despite waiting until radiographic studies had demonstrated that the leak had sealed.[54] In no case was extraluminal seeding of TCC demonstrated. There was only one reported case of ureteropelvic junction stricture in these 73 renal units, suggesting a lower overall incidence of stricture formation compared to ureteroscopic access to these pelvic tumors.

### ADJUVANT TOPICAL CHEMOTHERAPY/IMMUNOTHERAPY

The concern regarding extraluminal seeding associated with endoscopic management of upper tract TCC, as well as the polychronotopism inherent to the disease, have led to the increasingly frequent use of intracavitary topical therapy in the setting of upper tract TCC. Studer et. al. demonstrated the feasibility and efficacy of BCG used in the upper urinary tract.[60] Ten renal units with cytologic evidence for CIS were treated with BCG delivered via a percutaneous nephrostomy tube. Cytologies reverted to negative in 7/10 renal units. One patient developed BCG sepsis and required cessation of therapy. Since then, the safety and efficacy of intracavitary topical therapy in combination with endoscopic management in upper tract TCC has been demonstrated in many other studies.[30,31,39,46,48,50,52,53,55-57,61] The majority of investigators have favored the use of either BCG or mitomycin C. Access to the upper tract for topical therapy has been accomplished in a variety of ways, though the most reliable is via a nephrostomy tube left in place after percutaneous resection or fulguration. This permits reliable and reproducible exposure of the urothelium to the topical agent.

In the setting of ureteropyeloscopic management, the agent can either be delivered via a ureteral catheter or via bladder instillation after ureteral stent placement which purposely allows reflux to the upper tracts. However, the ability of the latter technique to provide adequate exposure of the urothelium to the topical agent has not been well established. Patients with refluxing urinary diversions can receive BCG or mitomycin C via a catheter into the conduit. Herr reported on a novel approach in a patient with grade 3 renal pelvic TCC and CIS in a patient with an ectopic solitary kidney.[62] After open resection, a freely refluxing pyelovesical anastomosis was created. The patient received six weekly instillations of BCG and remained tumor free at 13 months follow-up. A similar approach was reported by Ramsey and Soloway, who created a freely refluxing system after autotransplantation of a solitary kidney with recurrent pyelocalyceal TCC.[63]

The results achieved with intracavitary topical therapy have varied. Some investigators have shown some advantage to the addition of BCG or mitomycin C after endoscopic treatment of upper tract TCC.[30,48] For example, Orihuela and Smith found an 80% recurrence rate among patients who did not receive supplemental BCG versus a 16.6% recurrence rate among those who did.[48] In an update of their series however, no survival advantage to the addition of supplemental BCG could ultimately be demonstrated.[53] Martinez-Pineiro et. al. reported a 50% recurrence rate among patients with grade 2/3 or 3/3 disease not treated with supplemental therapy, versus a 27.7% recurrence rate among a similar group of patients who did receive some form of supplemental therapy.30 A variety of different supplemental therapies were tried in their study, including BCG, mitomycin C, thiotepa, 5-fluorouracil with uracil, and interferon-a2. Of these agents, the two most promising were BCG and mitomycin C.

In contrast, other studies have not demonstrated a significant difference between patients treated with adjuvant BCG or Mitomycin C versus those who had endoscopic treatment alone.[31,39] The variability among studies is likely due to the nonrandomized methods employed, and the tendency to treat either all patients with adjuvant therapy or only those at highest risk for recurrence or disease progression. To date, the true efficacy of adjuvant topical therapy awaits the results of prospective randomized studies.

Topical instillation of BCG or mitomycin C in the upper urinary tract is generally well tolerated. Perhaps most importantly, renal function has not been impaired after instillation of these agents. Persistent fever after BCG administration was reported in 3 of 59 (5.1%) patients in combined major series, though this resolved with appropriate antimicrobial therapy in all cases.[30,31,39,52,53,56] Up to 25% of patients may have granulomatous involvement of the upper urinary tract after BCG administration, however the significance of these findings is not clear.[64] Complications from the administration of topical immunotherapy/chemotherapy in the upper urinary tract can be avoided by maintaining low intracavitary pressures during administration. Furthermore, obstruction and extravasation should be ruled out with antegrade or retrograde radiographic studies prior to the instillation of these agents. At least one case of toxic agranulocytosis after absorption of extravasated mitomycin C has been reported.[30]

Recently the utility of oral bropirimine has been described in presumptive CIS of the upper urinary tract.[65] Sarosdy et. al. reported on 24 patients with positive cytologies and a negative radiographic and cystoscopic evaluation diagnosed as presumptive upper tract CIS. Ten (48%) of 21 patients converted to negative cytologies after treatment with oral bropirimine. Three patients dropped out of the study secondary to side effects which included rash, nausea and vomiting, and severe bone pain. Two of the ten patients who initially converted to negative cytologies later reverted to positive cytologies at 12 and 24 weeks after treatment. The role of bropirimine in patients undergoing endoscopic management of upper tract urothelial tumors remains to be determined, but clearly this has some potential efficacy.

### SUMMARY

The expanding experience with endourologic techniques in the setting of upper tract urothelial malignancy has demonstrated its safety and efficacy in carefully selected patients. Diagnostic accuracy is greatly enhanced and pathologic confirmation of disease is now possible. In carefully selected patients with low-grade and low stage disease, the results of endoscopic management have been especially encouraging. Patients with an anatomic or functionally solitary kidney, bilateral disease, or significant renal insufficiency who develop upper tract TCC should be considered for endourologic treatment as first line therapy. The role for endourologic management in the setting of low-grade, low stage disease in patients with a normal contralateral kidney remains controversial and awaits further study. Adjuvant topical therapy with BCG or mitomycin C appears safe and well tolerated after endourologic management of upper tract TCC. Its role in the setting of high-grade multifocal disease or CIS is promising, but determining the ultimate utility of these treatments will require prospective randomized trials.

### REFERENCES

1. Peterson, R.O.: Renal pelvis. In: Urologic Pathology. Edited by L.A. Biello. Philadelphia: Lippincott, pp. 181-228, 1986.
2. Charbit, L., Gendreau, M.C., Mee, S., Cukier, J.: Tumors of the upper urinary tract: 10 years experience. J Urol., 146: 1243, 1991.
3. Shinka, T., Uekado, Y., Aoshi, H., Hirano, A. and Ohkawa, T.: Occurrence of uroepithelial tumors of the upper urinary tract after the initial diagnosis of bladder cancer. J Urol., 140: 745, 1988.
4. Catalona, W.J.: Urothelial tumors of the urinary tract. In: Campbellís Urology, 6th ed. Edited by P. C. Walsh, A. B. Retik, T. A. Stamey, and E. D. Vaughan. Philadelphia: W. B. Saunders Co., vol. 2, Chapt. 28, pp. 1094-1158, 1992.

5. Wallace, D.M.A., Wallace, D.M., Whitfield, H.N., Hendry, W.F. and Wickham, J.E.: The late results of conservative surgery for upper tract urothelial carcinomas. Br J Urol., 53: 537, 1981.
6. Ziegelbaum, M., Novick, A.C., Streem, S.B., Montie, J.E., Pontes, J.E. and Straffon, R.A.: Conservative surgery for transitional cell carcinoma of the renal pelvis. J Urol. 138: 1146, 1987.
7. Huffman, J.L.: Ureteroscopic management of transitional cell carcinoma of the upper urinary tract. Urol Clin North Am. 15: 419, 1988.
8. Abdel-Razzak, O. and Bagley, D.H.: The 6.9F semirigid ureteroscope in clinical use. Urol. 41: 45, 1993.
9. Grasso, M. and Bagley, D.H.: A 7.5/8.2 F actively deflectable, flexible ureteroscope: a new device for both diagnostic and therapeutic upper urinary tract endoscopy. Urol. 43: 435, 1994.
10. Malek, R.S., Aquilo, J.J. and Hattery, R.R.: Radiolucent filling defects of the renal pelvis: classification and report of unusual cases. J Urol. 114: 508, 1975.
11. Zincke, H., Aquilo, J.J., Farrow, G.M., Utz, D.C. and Khan, A.U.: Significance of urine cytology in the early detection of transitional cell cancer of the upper urinary tract. J Urol. 116: 781, 1976.
12. Farrow, G.M., Utz, D.C. and Rife, C.C.: Morphological and clinical observations of patients with early bladder cancer treated with total cystectomy. Cancer Res. 36: 2495, 1976.
13. Murphy, D.M., Zincke, H., and Furlow W.L.: Primary grade 1 transitional cell carcinoma of the renal pelvis and ureter. J Urol. 123: 629, 1980.
14. Sarnacki, C.T., McCormick, L.J., Kiser, W.S., Hazard, J.B., McLaughlin, T.C. and Belovich, D.M.: Urinary cytology and the clinical diagnosis of urinary tract malignancy: a clinicopathologic study of 1400 patients. J Urol. 106: 761, 1971.
15. Gill, W.B., Lu, C.T., and Thomsen, S.: Retrograde brushing: A new technique for obtaining histologic and cytologic material from ureteral, renal pelvic and renal caliceal lesions. J Urol. 109: 573, 1973.
16. Blute, R.D. Jr. and Gittes, R.F.: Renal brush biopsy: Survey of indications, techniques and results. J Urol. 126: 146, 1981.
17. Streem, S.B., Pontes, J.E., Novick, A.C., and Montie, J.E.: Ureteropyeloscopy in the evaluation of upper tract filling defects. J Urol. 136: 383, 1986.
18. Bagley, D.H., Huffman, J.L. and Lyon E.S.: Flexible ureteropyeloscopy: diagnosis and treatment in the upper urinary tract. J Urol. 138: 280, 1987.
19. Bagley, D.H. and Rivas, D.: Upper urinary tract filling defects: flexible ureteroscopic diagnosis. J Urol. 143: 1196, 1990.
20. Keeley, F.X., Kulp, D.A., Bibbo, M., McCue P.A., and Bagley, D.H.: Diagnostic accuracy of ureteroscopic biopsy in upper tract transitional cell carcinoma. J Urol. 157: 33, 1997.
21. Badalament, R.A., Bennett, W.F., Bova, J.G., Kenworthy, P.R., Wise, H.A. II, Smith, S. and Perez, J.: Computed tomography of primary transitional cell carcinoma of upper urinary tracts. Urol. 40: 71, 1992.
22. Planz, B., George, R., Adam, G., Jakse, G. and Planz, K.: Computed tomography for detection and staging of transitional cell carcinoma of the upper urinary tract. Eur Urol. 27: 146, 1995.
23. McCoy, J.G., Honda, H., Reznicek, M. and Williams, R.D.: Computerized tomography for detection and staging of localized and pathologically defined upper tract urothelial tumors. J Urol. 146: 1500, 1991.
24. Huffman, J.L., Bagley, D.H., Lyon, E.S., Morse, M.J., Herr, H.W. and Whitmore, W.F.: Endoscopic Diagnosis and treatment of upper-tract urothelial tumors. A preliminary report. Cancer. 55: 1422, 1985.
25. Heney, N.M., Nocks, B.N., Daly, J.J., Blitzer, P.H. and Parkhurst, E.C.: Prognostic factors in carcinoma of the ureter. J Urol. 125: 632, 1981.
26. Chesko, S.B., Gray, G.F. and McCarron, J.P.: Urothelial neoplasia of the upper urinary tract. In Pathology Annual, part 2. Edited by Sommers, S.C. and Rosen, P.P. New York: Appleton-Century-Crofts, pp. 127-153, 1981.
27. Tawfiek, E.R. and Bagley D.H.: Upper-tract transitional cell carcinoma. Urol. 50: 321, 1997.
28. Gerber, G.S. and Lyon, E.S.: Endourological management of upper tract urothelial tumors. J Urol. 150: 2, 1993.
29. Streem, S.B.: Ureteropyeloscopy for upper urinary tract diagnosis. Sem Urol 7: 30, 1989.
30. Martinez-Pineiro, J.A., Matres, M.J.G. and Martinez-Pineiro, L.: Endourological treatment of upper tract urothelial carcinomas: analysis of a series of 59 tumors. J Urol. 156: 377, 1996.
31. Elliot, D.S., Blute, M.L., Patterson, D.E., Bergstralh, E.J. and Segura J.W.: Long-term follow up of endoscopically treated upper urinary tract transitional cell carcinoma. Urol. 47: 819, 1996.
32. Schmeller, N.T. and Hofstetter, A.G.: Laser treatment of ureteral tumors. J Urol. 141: 840, 1989.
33. Schilling, A., Bowering, R. and Keiditsch, E.: Use of the Neodymium-YAG laser in the treatment of ureteral tumors and urethral condylomata acuminata. Eur Urol. 12: suppl 1, 30, 1986.
34. Carson III, C.C.: Endoscopic treatment of upper and lower urinary tract lesions using lasers. Sem Urol. 9: 185, 1991.
35. Smith Jr., J.A., Lee, R.G. and Dixon, J.A.: Tissue effects of neodymium:YAG laser photo-radiation of canine ureters. J Surg Oncol. 27: 168, 1984.
36. Blute, M.L. and Patterson, D.E.: Current management of upper urinary tract radiographic filling defects. AUA update series. 15: 134, 1996.

37. Johnson, D.E.: Use of the homium:YAG (Ho:YAG) laser for treatment of superficial bladder carcinoma. Lasers Surg Med. 14: 213, 1994.
38. Matsuoka, K., Iida, S., Nakanami, M., Koga, H., Shimada, A., Mihara, T. and Noda, S.: Holmium: yttrium-aluminum-garnet laser for endoscopic lithotripsy. Urol. 45: 947, 1995.
39. Keeley Jr., F.X., Bibbo, M. and Bagley, D.H.: Ureteroscopic treatment and surveillance of upper urinary tract transitional cell carcinoma. J Urol. 157: 1560, 1997.
40. Johnson, D.E.: Treatment of distal ureteral tumors using endoscopic argon photoirradiation. Lasers Surg Med. 12: 490, 1992.
41. Tomera, K.M., Leary, F.J and Zincke H.: Pyeloscopy in urothelial tumors. J Urol. 127: 1088, 1982.
42. Lim, D.J., Shattuck, M.C. and Cook, W.A.: Pyelovenous lymphatic migration of transitional cell carcinoma following flexible ureteroscopy. J Urol. 149: 109, 1993.
43. McCarron, J.P., Mills, C. and Vaughan Jr., E.D.: Tumors of the renal pelvis and ureter: Current concepts and management. Sem Urol. 1: 75, 1983.
44. Kulp, D.A. and Bagley, D.H.: Does flexible ureteropyeloscopy promote local recurrence of transitional cell carcinoma?. J Endourol. 8: 111, 1994.
45. Andrews, P.A. and Segura, J.W.: Renal pelvic explosion during conservative management of upper tract urothelial cancer. J Urol. 146: 407, 1991.
46. Streem, S.B. and Pontes, E.J.: Percutaneous management of upper tract transitional cell carcinoma. J Urol. 135: 773, 1986.
47. Woodhouse, C.R.J., Kellett, M.J. and Bloom, H.J.: Percutaneous renal surgery and local radiotherapy in the management of renal pelvic transitional cell carcinoma. Brit J Urol. 58: 245, 1986.
48. Orihuela, E. and Smith, A.D.: Percutaneous treatment of transitional cell carcinoma of the upper urinary tract. Urol Clin N Amer. 15: 425, 1988.
49. Nolan, R.L., Nickel, J.C. and Froud, P.J.: Percutaneous endourologic approach for transitional cell carcinoma of the renal pelvis. Urol Radiol. 9: 217, 1988.
50. Blute, M.L., Segura, J.W., Patterson, D.E., Benson Jr, R.C. and Zincke H.: Impact of endourology on diagnosis and management of upper urinary tract urothelial cancer. J Urol. 141: 1298, 1989.
51. Tasca, A. and Zattoni, F.: The case for a percutaneous approach to transitional cell carcinoma of the renal pelvis. J Urol. 143: 902, 1990.
52. Vasavada, S.P., Streem, S.B. and Novick, A.C.: Definitive tumor resection and percutaneous bacillus calmette-guerin for management of renal pelvic transitional cell carcinoma in solitary kidneys. Urol. 45: 381, 1995.
53. Jarrett, T.W., Sweetser, P.M., Weiss, G.H. and Smith, A.D.: Percutaneous management of transitional cell carcinoma of the renal collecting system: 9-year experience. J Urol. 154: 1629, 1995.
54. Patel, A., Soonawalla, P., Shepherd, S.F., Dearnaley, D.P., Kellett, M.J. and Woodhouse, C.R.J.: Long-term outcome after percutaneous treatment of transitional cell carcinoma of the renal pelvis. J Urol. 155: 868, 1996.
55. Tasca, A., Zattoni, F., Garbeglio, A., Villi, G., Bassi, P. and Meneghini, A.: Endourologic treatment of transitional cell carcinoma of the upper urinary tract. J Endourol. 6: 253, 1992.
56. Schoenberg, M.P., Van Arsdalen, K.N. and Wein, A.J.: The management of transitional cell carcinoma in solitary renal units. J Urol. 146: 700, 1991.
57. Nakada, S.Y. and Clayman, R.V.: Percutaneous electrovaporization of upper tract transitional cell carcinoma in patients with functionally solitary kidneys. Urol. 46: 751, 1995.
58. Huang, A., Low, R.K. and White, R.D.: Nephrostomy tract tumor seeding following percutaneous manipulation of a ureteral carcinoma. J Urol. 153: 1041, 1995.
59. Streem, S.B.: Percutaneous management of upper-tract transitional cell carcinoma. Urol Clin N Amer. 22: 221, 1995.
60. Studer, U.E., Casanova, G., Kraft, R. and Zingg, E.J.: Percutaneous bacillus calmette-guerin perfusion of the upper urinary tract for carcinoma in situ. J Urol. 142: 975, 1989.
61. Eastham, J.A. and Huffman, J.L.: Technique of Mitomycin C instillation in the treatment of upper urinary tract urothelial tumors. J Urol. 150: 324, 1993.
62. Herr, H.W.: Durable response of a carcinoma in situ of renal pelvis to topical Bacillus Calmette-Guerin. J Urol. 134: 531, 1985.
63. Ramsey, J.C. and Soloway, M.S.: Instillation of Bacillus Calmette-guerin into the renal pelvis of a solitary kidney for the treatment of transitional cell carcinoma. J Urol. 143: 1220, 1990.
64. Bellman, G.C., Sweetser, P. and Smith, A.D.: Complications of intracavitary bacillus calmette-guerin after percutaneous resection of upper tract transitional cell carcinoma. J Urol. 151: 13, 1994.
65. Sarosdy, M.F., Pisters, L.L., Carroll, P.R., Benson, M.C., Moon, T.D., Lamm, D.L., Hudson, M.A., Lerner, S.P., Koch, M.O. and Schellhammer, P.F.: Bropirimine immunotherapy of upper urinary tract carcinoma in situ. Urol. 48: 28, 1996.

**This article should be referenced as follows:**

Clark, P.E. and Streem, S.B. (2004) Endourologic management of upper tract transitional cell carcinoma. *TheScientificWorldJOURNAL* **4 (S1)**, 62–75.

**Handling Editor:**

Anthony Atala, Principle Editor for *Urology* — a domain of *TheScientificWorldJOURNAL*.
